# Plant antimicrobials: extraction, characterization and activity against foodborne microorganisms

**DOI:** 10.1007/s12223-025-01417-7

**Published:** 2026-01-15

**Authors:** Fabiana França, Júlia Campos de Souza, Paula M. O’Connor, Andreia Pereira Matos, Natan de Jesus Pimentel-Filho

**Affiliations:** 1 Laboratory of Food Microbiology Advanced Studies (FoMAS), Centro de Ciências da Natureza, Universidade Federal de São Carlos, Rodovia Lauri Simões de Barros, Km 12 – SP189, Buri, 18290-000 SP Brazil; 2 Centro de Ciências e Tecnologias para a Sustentabilidade, Universidade Federal de São Carlos, Rodovia João Leme dos Santos, Km 110 – SP-264, Sorocaba, 18052-780 SP Brazil; 3 Teagasc Food Research Centre, Moorepark, Fermoy, Co. Cork, P61 C996 Ireland; 4 Centro de Ciências da Natureza, Universidade Federal de São Carlos, Rodovia Lauri Simões de Barros, Km 12 – SP189, Buri, 18290-000 SP Brazil

**Keywords:** Plant-derived antimicrobials, Foodborne pathogens, Antimicrobial peptides, Phenolic compounds, In silico screening, Molecular docking, Food preservation, Bioactive compounds

## Abstract

Plant-derived antimicrobials have been extensively studied due to their strong activity against foodborne and spoilage microorganisms, as well as their availability from diverse and cost-effective natural sources. A wide range of bioactive plant compounds, including phenolics, essential oils, alkaloids, lectins, and antimicrobial peptides have demonstrated significant potential in controlling microbial contamination in food systems. This review uniquely integrates advances in the extraction, purification, and molecular characterization of plant extracts and their bioactive antimicrobial compounds, along with insights into their mechanisms of action and in silico discovery approaches. Among these diverse bioactives, phenolics, essential oils, and antimicrobial peptides have shown the most promising potential for food applications. Recent progress in molecular docking and molecular dynamics simulations has accelerated the identification and optimization of plant antimicrobials, revealing their possible roles in inhibiting quorum sensing and biofilm formation. Despite these advances, knowledge gaps remain regarding their safety, stability, and interactions within complex food matrices, which must be addressed for industrial application. Overall, this review highlights both the opportunities and challenges in employing plant-derived antimicrobials as sustainable alternatives to synthetic preservatives, aligning food safety with consumer demand for natural products.

**Graphical abstract:**

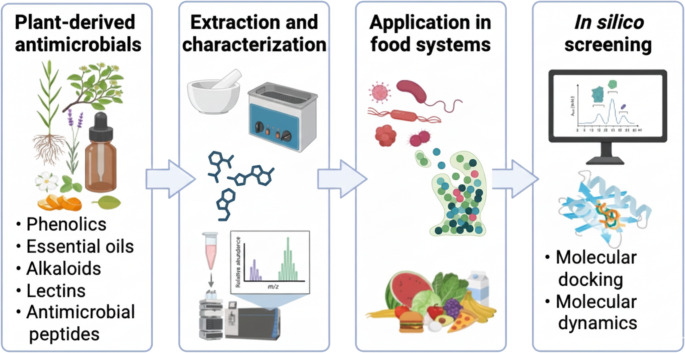

## Introduction

In recent years, the search for new antimicrobial agents and the evaluation of their bioactivity in diverse matrices has gained significant attention, particularly in response to the growing threat of drug-resistant pathogens. Among the key applications of antimicrobials is their role in improving food preservation by inhibiting undesirable microorganisms, including foodborne pathogens and spoilage organisms.

Rising consumer demand for natural, fresh, and additive-free food products has contributed to a shift away from synthetic preservatives and toward plant-derived compounds, which are naturally occurring, cost-effective, widely accessible, and generally recognized as safe (Lavilla and Gayán 2018; Martins et al. 2019). Notably, phytochemicals and plant-based bioactives have demonstrated potent antimicrobial activity against a wide spectrum of microorganisms, including bacteria, fungi, viruses, and protozoa (Chandra et al. 2017; Khameneh et al. 2019).

This review provides a comprehensive overview of plant-derived antimicrobials, emphasizing their botanical sources, molecular characteristics, and potential applications in the food industry. It also summarizes recent advances in innovative techniques for the extraction, purification, and characterization of these bioactive compounds, as well as the growing use of in silico tools, such as molecular docking and dynamics, for the analysis and optimization of phytochemicals. The manuscript was developed as a narrative synthesis of recent peer-reviewed studies retrieved from databases including Scopus, ScienceDirect, and PubMed, focusing on literature addressing plant compounds with antimicrobial activity and their technological applications.

## Plant antimicrobials: classification of antimicrobial secondary metabolites from plant sources

Plants produce a wide range of natural compounds, generally classified as primary or secondary metabolites. While primary metabolites are directly involved in vital functions, secondary metabolites serve mainly as protective agents, enabling plants to cope with adverse environmental conditions. These compounds are synthesized as part of adaptive responses to biotic and abiotic stress and exhibit far greater chemical diversity than primary metabolites (Saini et al. 2024). Medicinal plants, in particular, are recognized for their ability to produce molecular compounds capable of treating microbial infections and preserving food by inactivating the microorganisms responsible for spoilage and contamination (Chandra et al. 2017). They are rich in bioactive metabolites such as phenolics, alkaloids, lectins, and antimicrobial peptides, which contribute to these inhibitory effects. Aromatic plants, a subgroup of medicinal plants, produce fragrant antimicrobials such as essential oils, which have been traditionally used as natural preservatives in cereals, grains, pulses, fruits, and vegetables (Pandey et al. 2017).

**Fig. 1:**
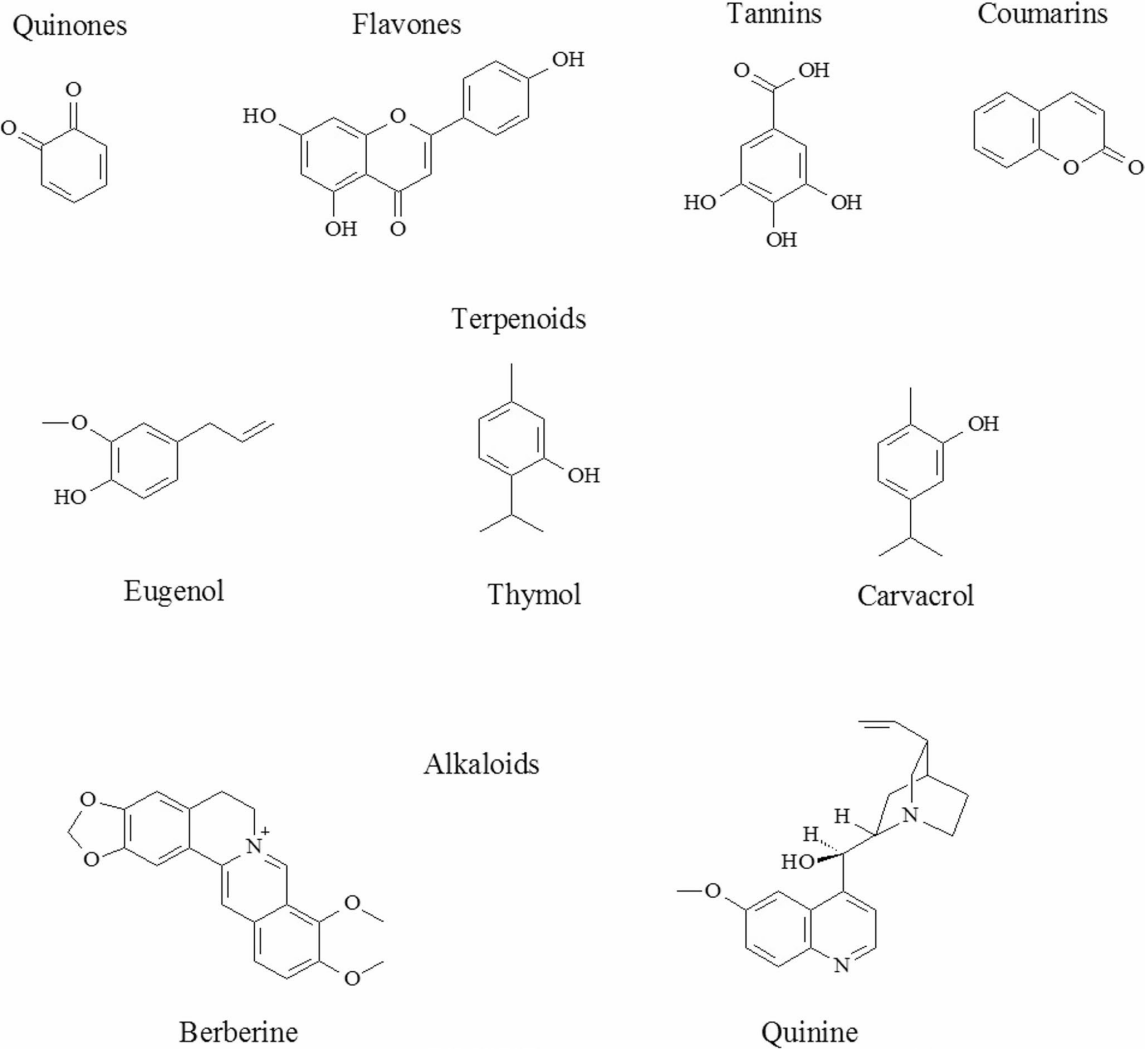
Representative chemical structures of the main classes of plant-derived bioactive compounds with antimicrobial activity

**Fig. 2:**
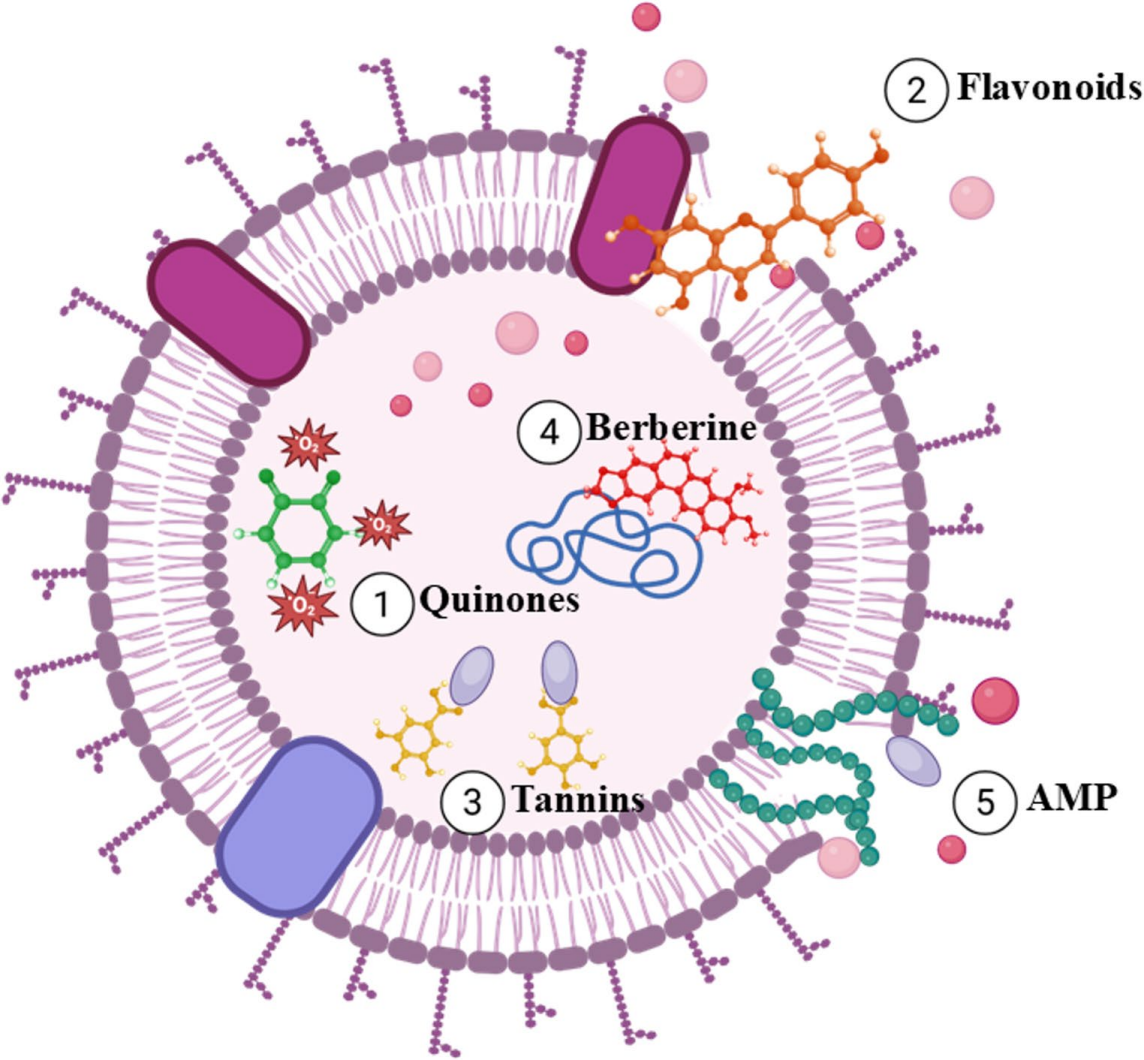
Mechanisms of antimicrobial action of plant-derived compounds. (1) Quinones act through redox cycling, generating reactive oxygen species that promote oxidative stress and destabilize microbial cells. (2) Flavonoids form complexes with extracellular and soluble proteins and destabilize microbial membranes. (3) Tannins inhibit essential microbial enzymes, disrupting key metabolic pathways. (4) Berberine interacts with DNA via an intercalative binding mode, as demonstrated by fluorescence, absorption, and competition assays. (5) AMPs interact electrostatically with the negatively charged bacterial surface, forming ion channels that lead to membrane disruption and cell lysis

**Phenolic compounds** are the most abundant group of secondary metabolites synthesized by plants and include quinones, flavonoids, tannins, and coumarins (Cowan 1999). These compounds typically contain one or more hydroxyl groups attached to an aromatic ring (Khadem and Marles 2010) (Fig. 1). Quinones, structurally defined as cyclohexadienediones with carbonyl groups positioned at 1,2 or 1,4 relative to each other (Ecevit et al. 2022), are potent antimicrobials that exert their effect through redox cycling, generating reactive oxygen species that cause oxidative stress and destabilize cells (Fig. 2) (Cowan 1999). They can also inactivate proteins by forming irreversible complexes with amino acids affecting surface adhesions, membrane enzymes, and polypeptides in the cell wall. Quinones in *Lawsonia inermis* (Family: Lythraceae), commonly known as henna, displayed antibacterial activity against *Pseudomonas aeruginosa*, an important pathogen responsible for food poisoning diseases and drinking water contamination (Othman et al. 2019). Flavonoids predominantly occur as glycosides in plants, while a smaller fraction is found in the form of free aglycones. Structurally, they possess a 2-phenylchromone core, consisting of two aromatic rings (A and B) connected by a three-carbon chain, often forming a heterocyclic ring (C), resulting in a C_6_–C_3_–C_6_ configuration. Based on the position of ring B, the oxidation level of the central chain, and the formation of additional rings, flavonoids are classified into several subclasses, including flavones, flavanones, isoflavones, flavonols, chalcones, flavanols, and anthocyanins (Chen et al. 2024). Flavonoids act as a defence mechanism in plants due to their ability to complex extracellular and soluble proteins and to disrupt microbial membranes (Fig. 2). Several studies have demonstrated their antibacterial, antiviral, and antifungal properties, the latter being able to affect *Aspergillus flavus* growth, whose mycotoxins may cause food intoxication (Chandra et al. 2017). Tannins are polymeric phenolic compounds widely distributed in plants. They are classified into two main categories: hydrolyzable and condensed tannins. Hydrolyzable tannins consist of gallic acid units esterified to D-glucose molecules. In contrast, condensed tannins, also known as proanthocyanidins, are more abundant and result from the polymerization of flavonoid monomers (Cowan 1999). Tannins exert their antimicrobial activity through different mechanisms, including direct interaction with microbial structures, inhibition of essential enzymes, and degradation of the cell membrane, ultimately leading to cell death (Fig. 2) (Huang et al. 2024). In previous studies, gallotannins, a type of hydrolyzable tannin, found in *Cytinus hypocistis* and *Cytinus ruber* (Family: Cytinaceae) extracts have shown antimicrobial and antibiofilm activities against three gram-positive bacteria, *Staphylococcus aureus*,* Staphylococcus epidermidis*, and *Enterococcus faecium* (Maisetta et al. 2019). Finally, coumarins are formed from benzene and oxygenated pyrone rings, and have documented antifungal and antibacterial activities against several pathogens (Al-Majedy et al. 2017). Propolis, a resinous substance produced by bees from plant exudates, containing polyphenols, phenolic aldehydes, quinones, and coumarins displays excellent antimicrobial activity as some of its antimicrobial components, are completely effective against *S. aureus* growth and partially effective against *E. coli* and *P. aeruginosa* (Harfouch et al. 2016).

**Essential oils** represent another group of secondary metabolites, largely made up of compounds with isoprene structures called terpenoids or terpenes (Fig. 1). These are the most abundant substances present in essential oils and are composed of five-carbon units (C_5_), also known as isoprene chains, which form the basis for their skeleton. They are classified into monoterpenes (C_10_), sesquiterpenes (C_15_), diterpenes (C_20_), triterpenes (C_30_), and tetraterpenes (C_40_), or carotenoids if they contain more than eight isoprene units. Their inhibitory properties are effective against bacteria, viruses, fungi, and protozoa (Cowan 1999). In a study to investigate the antimicrobial efficacy of essential oils, oxygenated terpenoids, such as eugenol, thymol, and carvacrol, were shown to have potential bactericidal activity against *Bacillus cereus*, *S. aureus*, *E. coli*, and *Salmonella enterica*, all pathogenic species related to food poisoning (Guimarães et al. 2019).

**Alkaloids** are characterized by the presence of heterocyclic nitrogen in their structure and are also known for their strong antimicrobial effects (Fig. 1). There are important representative components in this group, like berberine and quinines, that have been gaining attention in the research field for their potential activities against several pathogens. In an experimental study, berberine demonstrated significant inhibitory actions on the growth of *Streptococcus agalactiae*, a pathogenic bacterium commonly known to infect mammals and cause mastitis in dairy herds (Peng et al. 2015). Its mechanism is associated with its ability to interact with DNA, as fluorescence, absorption, and competition assays have shown that berberine binds to DNA through an intercalative mode (Fig. 2) (Li et al. 2012). In another study, quinines displayed inhibitory activity against *P. aeruginosa*, *Bacillus subtilis*, *S. aureus*, and *E. coli* (Antika et al. 2020).

**Lectins** are natural proteins or glycoproteins considered potent antimicrobials due to their ability to bind carbohydrates on microbial surfaces, resulting in cell agglutination. In plants, lectins play a key role in innate immunity and defense mechanisms, while in other organisms, they may also contribute to immune responses, cell signalling, apoptosis, and recognition processes in cell–cell interactions (Dias et al. 2015; Mishra et al. 2019). In a recent study, lectins isolated from fava bean (*Vicia faba*), lentil (*Lens culinaris*) and pea seeds (*Pisum sativum*), all from the Fabaceae family, were tested against several pathogenic bacteria and fungi and demonstrated inhibitory effects against *S. aureus* and *Streptococcus mutans* (gram-positive bacteria), *P. aeruginosa* and *Klebsiella pneumonia* (gram-negative bacteria), and *Candida albicans* (yeast) (El-Araby et al. 2020).

**Antimicrobial peptides (AMP)** are part of the innate immunity in plants and play an important role in their defense systems by killing invading pathogens and pests. AMP are a large class of peptides with diverse, antimicrobial activities. They can cause lysis in bacteria membrane cells through the interaction of their positive charge with the electronegative bacterial cell surface thereby creating ion channels (Fig. 2). After they enter the cell, they can also impede general synthesis through association with proteins, lipids, and nucleic acids. In addition to antibacterial effects, antifungal actions have also been reported (Cowan 1999; Farkas et al. 2017). AMP can block biofilm formation by both gram-positive and gram-negative bacteria. Some AMP also have antiviral and antifungal actions, especially against *Fusarium oxysporum*, *Mycosphaerella arachidicola*, *Physalospora piricola*, and *Botrytis cinereal* (Salas et al. 2015).

AMP Databases are web-based depositories that provide scientists with a readily accessible central source of reliable information on all known AMPs. The PhytAMP database includes peptide name, sequence, class, plant taxonomy, activity data (i.e. whether it is active against bacteria, fungi, viruses, or protozoa), structural determination method, and chemical properties (such as physicochemical and structural characteristics) (Hammami et al. 2009). More recently, PlantPepDB contains comprehensive information on almost 4000 plant-derived peptides including functional categorization, physicochemical properties, and activity against target microbes (Das et al. 2020).

The antimicrobials described above are the most commonly found in medicinal and aromatic plants and show the most promise in applications in both the food industry and pharmaceutical sectors.

## Main methods of extraction, purification, and characterization

### Extraction

Prior to developing useful applications, plant antimicrobials must pass through an extraction process to isolate the active components, purification to remove impurities, and molecular characterization to identify the type of bioactive agent. The first stage of this process consists of isolating the compound of interest from the source. Traditionally, compounds of interest were isolated by conventional methods including maceration, solvent extraction, and distillation whereas nowadays innovative methods are more desirable (Altemimi et al. 2017).

Solvents used to extract phytochemicals are chosen based on similar polarity to the bioactive compound, to ensure they properly dissolve the solute. The most common solvents used for this purpose are methanol, hexane, chloroform, acetone, ethyl acetate, and water. In an attempt to isolate antimicrobial and antioxidant compounds from *Prunus cerasoides* Buch-Ham. ex D. Don (Family: Rosaceae), the “Himalayan Wild Cherry”, maceration extraction was performed using different solvents. The highest yields were obtained with hydromethanol (6.2%) and methanol (5.8%). Among the tested extracts, the methanolic extract exhibited the strongest antibacterial activity, with the lowest MIC values (3.125 µg/mL) against *E. coli* and *S. aureus*. Other solvents, such as acetone and diethyl ether, demonstrated moderate to weak antibacterial effects, with MIC values ranging from 12.5 to > 50 µg/mL, depending on the bacterial strain (Agrawal et al. 2024). Solvent extraction is a relatively simple method but can be expensive and time consuming and the possibility of solvent traces in the extracts can affect their antimicrobial properties (Felhi et al. 2017). Apart from these disadvantages, solvent extraction is considered a reference method and is commonly used to compare the efficiency of different extraction techniques (Altemimi et al. 2017).

Aqueous extraction, the maceration of powdered plant material in water to promote the release of soluble compounds, followed by centrifugation to separate the solids, is an effective method for isolating phytochemical compounds. However, water is still rarely used for the extraction of antimicrobial compounds due to its high polarity, which limits the solubilization of less polar substances and generally leads to lower extraction yields. The antibacterial activity of plant extracts is frequently attributed to phenolic compounds, which may act through adsorption to cell membranes, enzyme inhibition, or chelation of essential nutrients such as substrates and metal ions (Abd El-Moaty et al. 2020). Solvent polarity plays a fundamental role in the extraction efficiency of phenolics and flavonoids, with higher yields generally associated with increased polarity, although excessively polar solvents may reduce extraction efficiency (Wakeel et al. 2019), such as water.

Studies have evaluated the effectiveness of different solvents in extracting antimicrobial compounds. Mehmood et al. (2022) investigated the use of aqueous, ethanol, and methanol solvents for the extraction of total phenolics, total flavonoids, antioxidant activity, and antibacterial activity from three medicinal plants found in Azad Kashmir (*Achillea millefolium*, *Bergenia ciliata*, and *Aloe vera*). The methanolic extract showed the strongest antibacterial activity against *S. aureus* and *E. coli*, which may be attributed to its higher total flavonoid content. Although the aqueous extract demonstrated notable antibacterial activity against both bacteria, it demonstrated the lowest overall yield. Another study that evaluated water, ethanol, and an ethanol: water mixture (50:50) as solvents in the maceration method demonstrated that solvent composition directly influences the antimicrobial activity of *Arum maculatum* extracts. The hydroethanolic extract showed the lowest MIC values against strains such as *E. coli* (25 mg/ml) and *S. aureus* (50 mg/ml), whereas the aqueous extract was the least effective, particularly against gram-negative bacteria. Overall, greater sensitivity was observed among gram-positive bacteria, such as *S. aureus* and *L. monocytogenes*, compared to gram-negative strains like *P. aeruginosa* and *E. coli*. This difference in susceptibility may be attributed to the structural characteristics of gram-negative cell walls, whose outer membrane acts as a barrier that hinders the penetration of active compounds, especially those with higher polarity (Farahmandfar et al. 2019).

Shen et al. (2002) proposed a buffer for extraction of proteinaceous compounds from plant material containing protease inhibitors such as ethylene glycol-bis (β-aminoethyl ether)-N, N,N′,N′-tetraacetic acid (EGTA) and phenylmethylsulfonyl fluoride (PMSF) and the reducing agent dithiothreitol (DTT). This buffer extracts and preserves the peptides and proteins for subsequent contaminant removal and protein concentration. In an experiment using a similar method, broccoli polypeptides were selectively extracted and then precipitated with ammonium sulphate and the aqueous extracts revealed potent antifungal properties and protease inhibitory activity. These activities found in broccoli water-soluble extracts are important to effectively reduce fungal contamination of cereals and to develop a promising food preservative in the future (Thery et al. 2020).

Innovative methods of extraction are of interest as they are faster, cheaper, and more environmentally friendly. Microwave-assisted extraction (MAE) represents an economical and efficient innovative method to reduce both extraction duration and solvent consumption. Initially, MAE increases the temperature and/or irradiation power, causing rupture of the cell wall and separation of the target solute from the active sites. This is followed by the diffusion of the solute from the plant matrix and the transference of the phytochemical components to the solvent system. This method was successfully used to extract polyphenolic compounds and oleuropein from olive leaves in a solvent-free reactor, and the method has proven more efficient than conventional extraction techniques (Ingle et al. 2017; Şahin et al. 2017).

Ultrasound assisted extraction (UAE), is an alternative method using sonification in an ultrasonic bath. Ultrasounds greater levels than 20 kHz, disrupt the plant cell walls and increase mass transfer, facilitating the movement of bioactive compounds and other phytochemicals from plant tissues into the solvent. This process increases solvent penetration and improves the diffusion of the target solutes, leading to higher extraction efficiency. UAE is considered a green technology as it has a lower impact on the environment due to reduced energy and solvent consumption (Talmaciu et al. 2015; Altemimi et al. 2017). In a comparative analysis between UAE and ethyl acetate solvent extraction methods for polyphenols from spruce bark, higher temperatures (60 °C) resulted in a higher yield than lower temperatures (45 °C), as the solubility increased as well as the diffusivity of the extracted chemicals. The study recommended UAE over conventional ethyl acetate extraction techniques, such as Soxhlet, due to better efficiency, faster processing times (5–60 min), purer extracts, and lower operating costs (Talmaciu et al. 2015).

Supercritical fluid extraction (SFE) operates with solvent properties over their critical points, when liquid and vapor can coexist at the highest pressure and temperature to isolate the bioactive components from the biomass. The procedure occurs inside a supercritical fluid extractor, where the gas is compressed into a dense liquid and then pumped to a chamber containing the material to extract. The gas is then recovered to use in future experiments. Carbon dioxide (CO_2_) is an ideal solvent as it is readily available, non-toxic, non-explosive, and residues are easy to remove and non-detrimental to biological properties of the molecular compounds of interest (Ingle et al. 2017). In the previously cited comparative analysis, using higher pressure (2500 psi) and lower temperature (35–50 °C) than UAE, SFE obtained similar quality extracts, with greater yield and purity. Although this method might have superior running costs, SFE is still recommended over classical solvent techniques, and even UAE, for the higher efficiency of extraction (Talmaciu et al. 2015). However, it is important to note that the applicability of SFE is closely related to the chemical characteristics of the target compounds. Due to the non-polar nature of supercritical CO₂, this method is particularly efficient for extracting non-polar or low-polarity substances such as lipids and essential oils (Khaw et al. 2017).

Pressurized liquid extraction (PLE) has emerged as an efficient and sustainable alternative to conventional solvent-based extraction methods, widely employed in the recovery of bioactive compounds from plants and food matrices. This technique involves a solid–liquid extraction process conducted at elevated temperatures (50–200 °C) and moderate pressures (10–15 MPa), conditions that keep the solvent in its liquid state even above its boiling point (Ramos et al. 2002). The combination of elevated pressure and high temperature enhances extraction kinetics and facilitates the desorption of bioactive compounds from the sample matrix while improving their solubility in green solvents such as ethanol and water (Višnjevec et al. 2024). In the study by Bagatini et al. (2023), PLE was applied to *Eugenia uniflora* L. leaves using water, ethanol, and a water: ethanol mixture to obtain phenolic-rich extracts. A total of 54 compounds were tentatively identified by LC-MS. The water: ethanol mixture yielded higher concentrations of phenolic acids and tannins, while ethanol extracts were richer in flavonoids. All extracts showed strong in vitro antioxidant activity and antibacterial effects, particularly against *S. aureu*s, reinforcing the potential of PLE as an effective method for selectively recovering bioactive compounds with multiple biological activities. Similarly, de Souza et al. (2025) demonstrated that PLE applied to yacon (*Smallanthus sonchifolius*) leaves under different solvent and temperature conditions led to increased recovery of phenolic compounds, enhanced antioxidant potential, and antimicrobial activity, including anti-quorum sensing effects, further supporting the versatility and efficiency of PLE for obtaining bioactive extracts from diverse plant matrices.

In summary, non-conventional methods are mostly cheaper, faster and demonstrate better yield and quality of extracts. They are also environmentally friendly due to reduced chemical usage. Therefore, innovative techniques have been extensively studied and developed in recent years. A comparative summary of the main advantages, limitations, and references related to the most commonly used extraction methods for plant-derived antimicrobials is presented in Table 1.

**Table 1:** Positive and negative aspects of the main extraction methods used for plant antimicrobials

Extraction method	Main characteristics	Advantages	Disadvantages	References
Maceration/Solvent extraction	Extraction using solvents such as methanol, ethanol, or hexane under ambient conditions	Simple and inexpensive; widely used reference method; suitable for thermolabile compounds	Time-consuming; high solvent consumption; possible solvent residue; low yield	Altemimi et al. (2017); Felhi et al. (2017); Zhang et al. (2018)
Aqueous extraction	Extraction using water as solvent	Safe, non-toxic, low cost, environmentally friendly	Low efficiency for non-polar compounds; low yield; not suitable for all phytochemicals	Abd El-Moaty et al. (2020); Breslow (2010)
Microwave-assisted extraction (MAE)	Uses microwave radiation to heat the solvent and plant matrix	Short extraction time; low solvent use; high efficiency; good reproducibility	Requires specific equipment; risk of thermal degradation of sensitive compounds	Ingle et al. (2017); Şahin et al. (2017); Cao et al. (2025)
Ultrasound-assisted extraction (UAE)	Uses ultrasound waves (> 20 kHz) to disrupt plant cells and enhance mass transfer	Green technology; faster and more efficient; reduced solvent and energy use	Possible oxidation or degradation due to cavitation; limited scalability	Talmaciu et al. (2015); Altemimi et al. (2017); Carreira-Casais et al. (2021)
Supercritical fluid extraction (SFE)	Uses fluids such as CO₂ above critical temperature and pressure	Produces solvent-free extracts; tunable selectivity; high purity and yield	High equipment cost; limited to non-polar or low-polarity compounds	Khaw et al. (2017); Ingle et al. (2017); Talmaciu et al. (2015)
Pressurized liquid extraction (PLE)	Extraction at high pressure and temperature, keeping solvent liquid	High efficiency; short extraction time; compatible with green solvents	Requires specialized equipment; not suitable for thermolabile compounds	Ramos et al. (2002); Višnjevec et al. (2024); Barp et al. (2023)

### Purification

Following extraction, a purification process is required to remove impurities and obtain a pure compound for precise antimicrobial activity assessment. The most common separation methods are column chromatography techniques, which consist of a solid stationary phase that separates compounds based on their size, charge, and hydrophobicity with the help of a liquid mobile phase (Ingle et al. 2017; Altemimi et al. 2017).

Ion exchange chromatography (IEC), is one of the simpler purification methods because the traveling rate depends directly on the polarity or charge of the material (Coskun 2016; Ingle et al. 2017). IEC effectively segregates molecules by charge, size, and shape, facilitating the separation of proteins, enzymes, and secondary metabolites like alkaloids and flavonoids in plant extracts. The process involves loading the extract onto a resin-packed column, followed by elution through pH or salt concentration adjustments. While IEC can be combined with other chromatographic techniques for enhanced purification, its high buffer costs and limitation to charged molecules are drawbacks. Nonetheless, it remains a widely used method for isolating plant-derived antimicrobial compounds (Yadav et al. 2024). Recent studies have demonstrated the effectiveness of ion-exchange chromatography in purifying AMPs from turmeric plant (*Curcuma longa* L). AMPs smaller than 3 kDa were isolated using cation-exchange and anion-exchange chromatography, followed by reversed-phase chromatography, leading to the selection of 12 peptides with significant antibacterial activity against *A. baumannii*, *S. epidermidis*, *S. aureus*, and *S. enterica* (Roytrakul et al. 2024). Another study utilized IEC to isolate antimicrobial peptides, specifically lipid transfer proteins, from *Capsicum chinense* seeds. These peptides exhibited fungistatic activity against *Candida albicans* and structural alterations in yeast cells (Oliveira et al. 2025).

Thin-layer chromatography (TLC) is a solid-liquid adsorption technique widely used for its simplicity, speed, low cost, and efficiency in identifying organic and inorganic compounds. The adsorbent material for the stationary phase consists of silica gel, alumina, or cellulose. The mobile phase, composed of one or more solvents, moves up the plate by capillary action, promoting the separation of sample components based on their polarity and solubility (Coskun 2016; Ingle et al. 2017). Its main advantages include low solvent consumption, fast processing, and easy detection. However, it has limitations, such as lower separation capacity for complex compounds and higher solvent consumption in automated processes (Yadav et al. 2024). A study by Agatonovic-Kustrin et al. (2022) used effect-directed analysis with TLC to screen antimicrobials in *Olea europaea* L. (‘Kalamata’) leaf extract. Maslinic and oleanolic acids were isolated using flash chromatography and exhibited significant activity against *Enterococcus faecalis*, *E. coli*, *S. mutans*, and *S. aureus*. Muwanya et al. (2024) used thin-layer chromatography-direct bioautography (TLC-DB) to isolate antimicrobial agents from *Capparis fascicularis* leaves. The hexane fraction exhibited the highest activity against *S. aureus* (MIC of 512 µg/mL), leading to the isolation of β-sitosterol using this technique.

Gas chromatography (GC) is a highly sensitive and selective technique used to separate and analyze volatile compounds in plant extracts. The process operates by partitioning between a gaseous mobile phase, typically helium, and a liquid stationary phase adsorbed onto an inert material. The sample is vaporized, injected into the chromatographic column, and transported by the carrier gas, separating based on volatility and interaction with the stationary phase. GC is widely applied in the analysis of essential oils, fragrances, and natural compounds, as well as in quality control of pharmaceuticals and pesticide detection. Its main advantages include high resolution, sensitivity, and rapid analysis. However, the technique is limited to volatile compounds and is not suitable for thermally unstable substances or those that interact with the column (Coskun 2016; Ingle et al. 2017; Yadav et al. 2024). A study by Siddique et al. (2025) utilized gas chromatography-mass spectrometry (GC-MS) to analyze the chemical composition of *Lavandula spica* essential oil, identifying 28 compounds, predominantly monoterpenes. The antibacterial activity was evaluated against *S. aureus* and *P. aeruginosa*, with the oil demonstrating greater efficacy against gram-positive bacteria.

High-performance liquid chromatography (HPLC) is a technique used to separate individual compounds from a mixture based on interactions between the stationary and mobile phases. High pressure pushes the extract through a column packed with adsorbent material, accelerating the purification process compared to simpler column chromatography (Ingle et al. 2017). The choice of column depends on the separation method: normal-phase HPLC uses a polar stationary phase, while reversed-phase HPLC uses a non-polar stationary phase (Mehata et al. 2022).

Modern equipment allows enhancements such as diode array detectors (DAD), which can measure the absorbance spectrum of analytes and provide additional spectral information from eluted compounds, although it does not directly impact purification efficiency (Ingle et al. 2017). In a recent study involving antimicrobial extraction, a liquid chromatographer with DAD, vacuum degasser, column oven, and automatic sampler, was used to separate phenolic compounds from solvents after maceration and to verify their purity, under 40 °C for 45 min. This technique was considered very sensitive, precise, robust and accurate for the experiment (Araújo et al. 2020).

These purification methods have been the most commonly applied to plant antimicrobial analysis in recent years, with a clear trend toward more optimized processes that offer shorter analysis times and higher-quality results. Among them, HPLC stands out for its high resolution, sensitivity, and ability to purify complex plant extracts efficiently. Additionally, GC is the preferred choice for volatile antimicrobial compounds, while TLC remains valuable for preliminary screening due to its simplicity and cost-effectiveness. Despite these advancements, traditional methods such as IEC continue to be useful for specific applications, such as purifying antimicrobial peptides, though they are more limited in scope (Yadav et al. 2024; Cetinkaya et al. 2025). Ultimately, as research on plant-derived antimicrobials expands, techniques that combine high selectivity, reproducibility, and scalability are likely to dominate due to their superior accuracy and adaptability for complex sample analysis. A comparative overview of the main purification techniques applied to plant-derived antimicrobial compounds is presented in Table 2, summarizing their key characteristics, advantages, and limitations.

**Table 2:** Positive and negative aspects of the main purification methods used for plant antimicrobials

Purification technique	Main characteristics	Advantages	Disadvantages	References
Ion-exchange chromatography (IEC)	Separation based on electrostatic interactions between charged analytes and oppositely charged groups on the resin	High selectivity for charged molecules; efficient for peptides, proteins, and alkaloids; compatible with aqueous systems	Limited to ionizable compounds; high buffer cost; requires pH and ionic strength optimization	Coskun (2016); Yadav et al. (2024); Rajabiyan et al. (2025)
Thin-layer chromatography (TLC)	Solid–liquid adsorption on a thin layer of silica, alumina, or cellulose; separation occurs by polarity and solubility differences	Simple, low-cost, rapid screening method; minimal solvent use; easy visualization	Lower resolution for complex mixtures; limited scalability; possible solvent waste in automated systems	Coskun (2016); Yadav et al. (2024); Zych and Pyka-Pająk (2025)
Gas chromatography (GC)	Separation of volatile compounds via partitioning between a gaseous mobile phase (e.g., helium) and a liquid stationary phase	High resolution and sensitivity; fast analysis; excellent for volatile compounds	Restricted to volatile and thermally stable compounds; requires derivatization for some analytes	Coskun 2016; Ingle et al. (2017); Yadav et al. (2024); Costa et al. (2010)
High-performance liquid chromatography (HPLC)	Separation of compounds under high pressure through a packed column; can operate in normal or reversed phase modes	High resolution and reproducibility; suitable for complex extracts; allows qualitative and quantitative analysis	Expensive equipment; solvent and maintenance costs; requires trained personnel	Ingle et al. (2017); Mehata et al. (2022); Araújo et al. (2020)

### Characterization

Once the compound has been purified it must be identified and characterized. Characterization plays a crucial role in determining the chemical composition of extracts, providing detailed information on their molecular structure, molecular mass, surface charge, size, and functional groups (Wong et al. 2023). Spectroscopy is a method that correlates electromagnetic radiation and the absorption of light. Basically, radiation is emitted through an organic molecule, so the amount of absorbed radiation, also called absorbance, is measured by a spectrophotometer, to produce a spectrum profile. From these spectra, it is possible to identify molecular structures. Spectroscopic techniques include UV-visible (UV-Vis), Infrared (IR), Nuclear Magnetic Resonance (NMR), and mass spectrometry (MS) (Altemimi et al. 2017).

MS is a powerful tool for identifying the molecular mass and structural characteristics of compounds within a sample. When integrated with chromatographic techniques, it provides a detailed chemical profile of plant extracts. Analyzing the structure of plant-based compounds is a challenging task that necessitates the use of multiple complementary analytical approaches (Yadav et al. 2024). This integration allows for both the purification and identification of phytochemicals in a single process. For instance, gas chromatography coupled with mass spectrometry (GC-MS) has been widely applied to isolate and chemically characterize the volatile constituents of *Thymus vulgaris* (Family: Lamiaceae) essential oils, which were subsequently evaluated for their antifungal activity in independent bioassays (Satyal et al. 2016). Similarly, GC-MS has been employed to identify volatile terpenes from tobacco leaves (*Nicotiana tabacum*; Family: Solanaceae) (Gao et al. 2018). Liquid chromatography systems are also routinely connected to mass detectors (LC-MS) to separate and identify metabolites in plant extracts by comparing the mass spectral information to curated databases. This method was used to identify the total polyphenolic content of cherry (*Prunus cerasus* L.; Family: Rosaceae) and blackcurrant (*Ribes nigrum* L.; Family: Grossulariaceae) leaf extracts, in an experiment designed to identify natural antioxidant and antimicrobial agents for meat products (Nowak et al. 2016).

UV spectroscopy is a widely used, non-destructive technique for analyzing plant extracts by measuring their absorption of ultraviolet light. It is particularly effective for detecting flavonoids, phenolic acids, and alkaloids, which exhibit characteristic absorption spectra in the UV range. This method helps identify and quantify compounds while also assessing extract purity, as impurities can cause additional peaks or broad absorption bands (Wong et al. 2023; Han et al. 2025). HPLC equipped with a UV-Vis detector was used to analyze the phenolic and flavonoid components of *Ficus carica* leaf extract. These compounds were linked to the extract’s antibacterial and antifungal activity, highlighting the effectiveness of HPLC-UV/Vis in characterizing plant-derived antimicrobials for agricultural applications (Lackner et al. 2025).

Spectroscopy can also be used individually, as demonstrated by the use of Fourier Transmission Infrared Spectroscopy (FTIR). FTIR is a non-destructive technique that analyzes infrared absorption to determine the chemical composition of plant extracts, identifying functional groups in compounds such as flavonoids and alkaloids. It provides a unique spectral fingerprint and requires minimal sample preparation, making it valuable for assessing extract quality, purity, and potential applications in natural product development (Felhi et al. 2017; Yadav et al. 2024). FTIR identified key functional groups in *Lavandula spica* essential oil, confirming monoterpenes as the main compounds linked to its antimicrobial activity against *S. aureus* and *P. aeruginosa* (Siddique et al. 2025). Similarly, FTIR analysis of *Calotropis procera* leaf extract detected hydroxyl, carbonyl, aldehyde, and phenol groups, which contribute to its therapeutic potential, particularly its antimicrobial effects against gram-positive bacteria (Adem Endris et al. 2024).

Nuclear Magnetic Resonance (NMR) is a quantitative technique routinely used to determine the structure of organic molecules at an atomic level. NMR works by placing a sample in a strong magnetic field and exposing it to radiofrequency radiation, causing certain atomic nuclei, such as hydrogen or carbon, to absorb energy. The resulting signals provide detailed information about the molecular structure and atomic environment (Altemimi et al. 2017; Han et al. 2025). In a study investigating natural preservatives, NMR was used to elucidate the chemical structure of luteolin, the key antimicrobial compound isolated from *Rumex tingitanus* leaf extract (Family: Polygonaceae). The ethyl acetate fraction showed the highest antimicrobial activity, leading to the isolation of luteolin, which effectively inhibited *Listeria monocytogenes* in minced beef in a concentration- and time-dependent manner, highlighting its potential as a natural preservative (Mhalla et al. 2017). Another study utilized NMR spectral analysis to characterize biflavonoid and amentoflavone, two metabolites isolated from *Nandina domestica* (Family: Berberidaceae), commonly known as heavenly bamboo. These compounds were later evaluated for their antioxidant and antibacterial properties, demonstrating efficacy both in vitro and in food matrices, including minced chicken and apple juice (Bajpai et al. 2019). In addition to its high sensitivity and non-destructive nature, NMR can determine the structure of various chemical species in a single experiment, particularly organic molecules in solution. It also helps analyze molecular interactions within extracts, enhancing the understanding of their biological activity. Overall, NMR is a powerful tool for characterizing plant extracts and optimizing natural product development (Rivero-Cruz et al. 2017; Yadav et al. 2024).

Matrix-Assisted Laser Desorption/Ionization Time-of-flight mass spectrometry (MALDI-TOF MS) is an efficient technique for identifying and characterizing bioactive compounds, offering greater speed and sensitivity compared to traditional methods. It has been widely used to analyze a variety of biomolecules, particularly peptides and proteins (Angeletti 2017). MALDI-TOF MS was used to profile plant antimicrobial peptides from *Calliandria portoriscensis* extracts (Family: Fabaceae), capable of inhibiting gram-positive and gram-negative bacterial growth (Ogbole et al. 2020). In addition, MALDI-TOF MS was used to determine the peptide sequences of AMPs from different plant sources, allowing the assessment of their bioactivity and enabling further investigation of these molecules in antimicrobial applications (Tang et al. 2018).

Circular dichroism (CD) spectroscopy measures the differential absorption of left- and right-circularly polarized light by chiral molecules, which absorb preferentially in one direction. This technique is particularly useful for analyzing the structural properties of plant-derived bioactive compounds, including antimicrobials. CD spectroscopy demonstrated great utility in the structural characterization of broccoli proteins and peptides by analyzing their spectra in deionized water and comparing them to known molecular conformations (Thery et al. 2020). Antimicrobial indole alkaloids from *Gelsemium elegans* (Family: Loganiaceae) had their structures elucidated by electronic CD calculation, leading to subsequent antimicrobial studies on the molecules (Wei et al. 2018).

Overall, there is no single method capable of extracting, purifying, and characterizing all phytochemicals, as each technique is suited to specific compounds and analytical goals. For instance, chromatography-based methods such as HPLC and GC are ideal for separating and identifying volatile and non-volatile compounds, while spectroscopic techniques like FTIR and UV-Vis excel in functional group analysis and purity assessment. Additionally, NMR provides detailed structural elucidation, whereas MALDI-TOF MS is particularly effective for characterizing peptides and proteins. The choice of technique depends on factors such as the nature of the phytochemical, the intended application, and the available experimental conditions. As research advances, new assays are continuously being developed to refine the study of plant-derived antimicrobial compounds, enhancing both efficiency and accuracy in their analysis (Tang et al. 2018; Araújo et al. 2020; Yadav et al. 2024). These analytical techniques collectively illustrate the complexity and precision required in the characterization of plant-derived antimicrobials. Their main analytical principles and comparative aspects are detailed in Table 3.

**Table 3:** Positive and negative aspects of the main characterization methods used for plant antimicrobials

Characterization techniques	Main characteristics	Advantages	Disadvantages	References
UV–Visible (UV–Vis) spectroscopy	Measures the absorption of ultraviolet and visible light by molecules; provides electronic transition data useful for identifying aromatic and conjugated systems	Simple, fast, non-destructive; allows qualitative and quantitative analysis; useful for phenolics, flavonoids, and alkaloids	Limited structural information; interferences from impurities; applicable mainly to chromophoric compounds	Han et al. (2025); Wong et al. (2023); Ríos-Reina and Azcarate (2023)
Fourier Transform Infrared (FTIR) spectroscopy	Detects molecular vibrations associated with functional groups by measuring infrared absorption; produces a spectral fingerprint	Non-destructive; minimal sample preparation; rapid identification of functional groups	Overlapping peaks can complicate interpretation; less effective for complex mixtures; qualitative rather than quantitative	Felhi et al. (2017); Yadav et al. (2024); Mondal et al. (2024)
Nuclear Magnetic Resonance (NMR) spectroscopy	Detects the magnetic properties of atomic nuclei in a magnetic field to determine molecular structure at the atomic level	Highly detailed structural elucidation; quantitative; non-destructive; suitable for organic molecules in solution	Requires large sample amounts and expensive equipment; not ideal for complex mixtures without prior purification	Altemimi et al. (2017); Han et al. (2025); Rivero-Cruz et al. (2017); Mondal et al. (2024)
Mass spectrometry (MS)	Determines molecular mass and structural fragments by ionizing compounds and measuring mass-to-charge ratios	High sensitivity and selectivity; provides molecular weight and fragmentation pattern; can be coupled to chromatography (GC-MS, LC-MS)	Requires ionizable and thermally stable compounds; possible matrix interferences	Yadav et al. (2024); Mondal et al. (2024)
Matrix-Assisted Laser Desorption/Ionization Time-of-Flight (MALDI-TOF MS)	Ionizes large biomolecules using a laser and measures their time-of-flight to determine molecular weight	Rapid, highly sensitive; ideal for peptides and proteins; fast data acquisition and easy operation	Ionization suppression of low-abundance analytes; sensitivity to sample impurities and contaminants; need for complex sample preparation and purification	Angeletti (2017); Dave et al. (2011)
Circular Dichroism (CD) spectroscopy	Measures the differential absorption of left- and right-circularly polarized light to determine chirality and secondary structure	Non-destructive; effective for studying conformations of chiral molecules, proteins, and peptides	Requires optically active samples; sensitive to buffer, solvent, and excipient composition; provides limited structural detail compared to NMR	Thery et al. (2020); Oyama et al. (2026)

## Plant antimicrobials: pathogen control and food system applications

Plant-derived antimicrobials serve as natural preservatives, offering the potential to enhance food safety and extend shelf life. Unlike synthetic preservatives, which may pose health risks due to residual toxicity, natural alternatives are often considered safer for consumption (Woo et al. 2023). Before testing the antimicrobial activity of plant extracts in food matrices, it is essential to first assess their effectiveness under in vitro conditions. Various microbiological assays are commonly employed for this purpose, including agar well diffusion, disk diffusion, and broth microdilution methods, which allow the determination of inhibition zones and minimum inhibitory concentrations (MICs) against target microorganisms (Klančnik et al. 2010; CLSI 2024). A study conducted by Malik et al. (2024) evaluated the antimicrobial potential of *Bismarckia nobilis*, *Choysia ternata*, *Chamaedorea cataractarum*, and *Beaucarnea recurvata* extracts using agar well diffusion and broth microdilution assays. The results demonstrated strong inhibitory effects against both Gram-positive and Gram-negative bacteria, highlighting the broad antimicrobial spectrum of these plant species. Similarly, Ngamsurach and Praipipat (2022) assessed the antibacterial activity of *Garcinia cowa* and *Piper sarmentosum* extracts through the disk diffusion method, which revealed distinct inhibition zones against *Staphylococcus aureus* and *Escherichia coli*. These preliminary evaluations are crucial for selecting the most active extracts or compounds for subsequent testing in food systems. Table 4 presents recent studies evaluating the effect of plant antimicrobial extracts on foodborne microorganisms in different food matrices.

**Table 4:** Application of plant extracts against foodborne microorganisms in food matrices

Species	Common name	Extraction/purification/ characterization methods	Major active component	Tested against	Food matrix	Reference
*Carya illinoinensis* (Wangenh) C. Koch	Pecan nutshell	Aqueous extraction	Essential oil	*L. monocytogenes*,* Salmonella* Enteritidis, *S. aureus*,* B. cereus*, *Aeromonas hydrophila*,* P. aeruginosa*	Lettuce	Caxambú et al. (2016)
*Rumex tingitanus*	*Koressa*	Maceration with aqueous ethanol extraction, TLC, NMR, UV, LC-MS	Total phenolics and flavonoids	*L. monocytogenes*	Minced beef meat	Mhalla et al. (2016)
*Achillea fragrantissima*,* Salvia officinalis*,* Thymus vulgaris*,* Rosmarinus officinalis*	Achillea, rosemary, sage, and thyme	Hydrodistillation, GC-MS	Essential oil	*E. coli*	Juice	Hatab et al. (2016)
*Schinus terebinthifolius* Raddi	Pink pepper tree	Hydrodistillation, GC-MS	Essential oil	*L. monocytogenes*	Minas cheese	Dannenberg et al. (2016)
*Mentha pulegium*	-	Hydrodistillation, GC-MS	Essential oil	*L. monocytogenes*	Iranian white cheese	Sadeghi et al. (2016)
*Citrus lemon*	Lemon	Steam distillation, GC-MS	Essential oil	*E. coli*,* L. monocytogenes*,* P. aeruginosa*,* Salmonella enterica*,* S. aureus*,* Aspergillus* spp., *Fusarium spp.*	Minced beef meat	Ben Hsouna et al. (2017)
*Cinnamomum javanicum*	-	Acetone: methanol: water extraction, UV-VIS, GC-MS	Volatile components	*L. monocytogenes*	Smoked salmon	Yuan et al. (2017)
*Carthamus tinctorius* L.	Safflower seed	Ethanol extraction	Essential oil	*Salmonella* Typhimurium, *E. coli*,* L. monocytogenes*,* S. aureus*	Lettuce	Son et al. (2017)
*Vaccinium oxycoccos* L.	Swamp cranberry	Aqueous ethanol extraction, HPLC	Organic acids, flavonols, terpenes and stilbenes	*S. aureus*,* L. monocytogenes*,* Salmonella* Enteritidis, *E. coli*	Minced pork meat	Stobnicka and Gniewosz (2018)
*Satureja hortensis* L.	Summer savory	Ethanol extraction, GC-MS	Terpenes	*S. aureus*	Cheese	Alexa et al. (2018)
*Nigella sativa*	Black cumin	-	Essential oil	*E. coli*,* S. aureus*	Cheese	Georgescu et al. (2018)
*Nandina domestica*	Heavenly bamboo	NMR	Biflavonoid, amentoflavone	*E. coli*,* S. aureus*	Minced chicken meat and apple juice	Bajpai et al. (2018)
*Zingiber officinale* Roscoe	Ginger	Hydrodistillation, GC-MS, FTIR	Essential oil encapsulated	*L. monocytogenes*,* S. aureus*,* E. coli*,* Salmonella* Typhimurium, *P. aeruginosa*	Minas cheese	da Silva et al. (2018)
*Syzygium antisepticum*	-	Acetone: methanol: water extraction, UV-VIS, GC-MS	Volatile components	*S. aureus*	Cooked chicken	Yuan and Yuk (2018)
*Citrus limon* var. *pompia* Camarda	Sardinian lemon	Steam distillation, GC-MS	Gaseous essential oil	*L. monocytogenes*	Ricotta cheese	Fancello et al. (2020)
*Tetrapleura tetraptera*	-	Ethanolic extraction, GC-MS	Citral	*E. coli* and *S. aureus*	Pork meat	Lin et al. (2019)
*Origanum vulgare*,* Thymus vulgaris*,* Cinnamomum verum*	Oregano, thyme and cinnamon	Steam distillation, GC-MS	Cellulosic pads	*P. putida*,* P. fragi*,* P. fluorescens*,* Enterococcus faecalis*,* Lactococcus lactis*,* Salmonella enterica*,* Campylobacter jejuni*,* S. aureus*	Minced beef meat	Agrimonti et al. (2019)
*Melaleuca alternifolia*	Tea tree	Steam distillation, GC-MS	Terpinen-4-ol	*L. monocytogenes*	Ground beef	Silva et al. (2019)
*Passiflora cincinnata* Mast.	Passion fruit	Aqueous extraction	Phenolics, terpenoids and flavonoids	*Listeria* spp., *S. aureus*	Coalho cheese	Costa et al. (2020)
*Cudrania tricuspidata*	Silkworm thorn	Ethanol extraction, SDS-PAGE	Phenolic compounds	*L. monocytogenes*,* E. coli*,* Salmonella* Typhimurium	Romaine lettuce and kale	Woo et al. (2020)
*Origanum vulgare*,* Thymus vulgaris*,* Rosmarinus officinalis*,* Laurus nobilis*,* Mentha spicata*,* Calendula officinalis*,* Ocimum basilicum*,* Zea mays silk*	Oregano, Thyme, Rosemary, Bay leaf, Mint, Calendula, Basil, Corn silk	Hydrodistillation; UPLC-HESI-MS/MS; HPLC-DAD	Phenolic acids (rosmarinic, chlorogenic), flavonoids (hesperidin, luteolin), carvacrol, thymol	*Salmonella* Typhimurium	Pork meat (marinated)	Gavriil et al. (2021)
*Vaccinium macrocarpon* and *Punica granatum L.*	Cranberry and Pomegranate	Aqueous and ethanolic extraction; LC-QTOF/MS (extracts); GC/MS (essential oils)	Phenolic acids (quinic, chlorogenic), citric acid, p-cymene, carvacrol, thymol	*Enterobacteriaceae*, mesophilic bacteria, yeasts/molds, *Staphylococcus* spp., *Pseudomonas* spp., lactic acid bacteria	Pork meatballs	Mantzourani et al. (2022)
*Colocasia esculenta*	Taro leaf	Aqueous extraction, HPLC (polyphenols); GC-MS (volatiles)	Polyphenols, flavonoids, sulfated polysaccharides, triterpenoids, tannins	*Salmonella enterica*,* L. monocytogenes*	Fermented milk beverages	Shehata et al. (2023)
*Salvia officinalis*,* Lavandula stoechas*,* Artemisia dracunculus*,* Hyssopus officinalis*	Sage, Lavender, Tarragon, Hyssop	Steam distillation (essential oils); ethanolic extraction; GC-FID; HPLC-DAD	Caffeic, protocatechuic, syringic acids; cineole, terpineol, methyl eugenol	*Clostridium tyrobutyricum*	Hard cheese	Ávila et al. (2023)
*Cymbopogon citratus* and *Laurus nobilis*	Lemongrass and Bay leaf	Aqueous and ethanolic extraction, phytochemical screening (qualitative)	Tannins, flavonoids, mucilage, alkaloids	*E. coli*,* Staphylococcus* spp., lactic acid bacteria	Fermented cereal gruel (Benin)	Karimou et al. (2024)
*Cynara scolymus*	Artichoke leaf	Ethanolic extraction; HPLC (polyphenols); GC-MS (volatiles)	Phenolic acids, flavonoids	*L. monocytogenes*,* L. innocua*,* S. aureus*,* B. cereus*,* S. enterica*,* E. coli*,* P. aeruginosa*	Soft cheese	Mahgoub et al. (2025)

When comparing the performance of plant-derived antimicrobials across food matrices, multiple intrinsic and extrinsic factors substantially influence their efficacy. In high-fat and high-protein foods, EOs often exhibit reduced activity because their hydrophobic constituents preferentially partition into the lipid phase, while proteins and carbohydrates can bind phenolic compounds and form less active complexes, thereby limiting diffusion and decreasing antimicrobial potency (Oulahal and Degraeve 2022; Zheng et al. 2024; Gómez-Llorente et al. 2025). Similar matrix-dependent interactions have been reported for antimicrobial peptides, whose activity can be reduced by changes in pH, ionic strength, temperature, or the presence of metal ions, all of which alter peptide charge, conformation, and membrane binding (Nagella et al. 2025). Water activity further modulates both microbial susceptibility and the stability or mobility of antimicrobials within the food matrix (Wang et al. 2024). These combined effects contribute to the well-documented gap between low MIC values obtained in vitro and the considerably higher concentrations required for effective in situ control in real foods. Nevertheless, when properly formulated or delivered, plant-derived antimicrobials can still result in meaningful shelf-life extension, particularly in high-moisture matrices where microbial proliferation occurs more rapidly.

Fungal growth leads to commodity deterioration and widespread food contamination. Mycotoxin production by fungi often triggers lipid oxidation, resulting in significant agro-industrial losses and posing a food safety risk to humans upon ingestion. To address this issue, essential oils have been explored as natural antifungal agents. For example, ajowan essential oil, rich in thymol and derived from *Trachyspermum ammi* (Family: Apiaceae), exhibited strong antifungal, antiaflatoxigenic, and antioxidant activity against *Aspergillus flavus*, effectively protecting food commodities for up to 12 months of storage (Kedia et al. 2015). Similarly, water-soluble extracts of broccoli seeds (*Brassica oleracea* var. *italica*; Family: Brassicaceae) inhibited *Fusarium culmorum* and *Aspergillus niger* growth in vitro. At higher concentrations, these extracts completely prevented food spoilage caused by *F. culmorum* in barley grains (Thery et al. 2020). In meat preservation studies, white mustard essential oil, derived from *Sinapis alba* L., combined with carvacrol, exhibited inhibitory effects against *Salmonella* both in vitro and in ground chicken meat over a 12-day storage period (Jabeen and Khanum 2017; Porter et al. 2020).

Proteinaceous antimicrobials also show promise as food preservatives. Antimicrobial peptides isolated from *Momordica charantia* L. (Family: Cucurbitaceae), commonly known as bitter melon, were tested against common foodborne pathogens. Crude extracts significantly reduced bacterial growth in vitro; however, their antibacterial effects were enhanced after purification, particularly against *S. aureus* and *E. coli*. To evaluate the potential of the purified peptide as a bio-preservative, *S. aureus* was inoculated into minced meat treated with different extract concentrations. At 40 µg peptide concentration, viable *S. aureus* counts decreased significantly from 8.0 to 3.77 log CFU (*P* < 0.001), demonstrating a dose-dependent inhibitory effect. Although further research is needed to confirm its long-term efficacy and assess its effects on other microorganisms, antimicrobial peptides from *M. charantia* L. show strong potential as natural bio-preservatives for minced meat products (Jabeen and Khanum 2017).

Olive leaf extracts rich in phenolic compounds have also been investigated for their role in poultry meat preservation. These extracts extended the shelf life of raw chicken meat by six days compared to non-phenolic controls under refrigerated conditions (4–10 °C). This treatment exhibited antioxidant and antibacterial activities against foodborne pathogens while maintaining sensory attributes (Saleh et al. 2020). Similarly, a phenolic extract from *Hibiscus sabdariffa* (rosella; Family: Malvaceae) exhibited antibacterial activity in vitro and extended the shelf life of sprayed beef meat by six days compared to untreated meat. Additionally, being derived from a Generally Recognized as Safe (GRAS) flower, the phenolic extract did not alter the cooked meat’s color and enhanced its flavor with a pleasant taste (Panda et al. 2019; Márquez-Rodríguez et al. 2020).

The antimicrobial potential of edible plants in combating foodborne pathogens has been extensively studied. Forty-six species of Indian plants, rich in phenols, flavonoids, tannins, saponins, steroids, and terpenes, were selected based on their well-known antimicrobial properties. Most of the crude extracts tested inhibited both gram-positive (*B. cereus*,* Streptococcus faecalis*,* Listeria innocua*,* Micrococcus luteus*) and gram-negative (*E. coli*,* P. aeruginosa*,* S. enterica*,* Shigella sonnei*) bacteria, with gram-positive strains being more susceptible. These findings suggest that these plants could serve as potential alternatives for controlling foodborne pathogens and, with further research, may be developed as effective antimicrobial additives in food matrices (Panda et al., 2019). A similar investigation screened approximately 800 plant species from various countries to identify extracts capable of inactivating *L. monocytogenes* and preventing related foodborne illnesses. Twelve plant extracts effectively inhibited *L. monocytogenes* growth and were identified as promising natural preservatives (Ceruso et al. 2020).

This review highlights some of the most significant research on plant antimicrobials as natural food preservatives. While numerous studies have been conducted in this field, those discussed here represent key advancements in understanding and applying plant-derived antimicrobial compounds. Antimicrobial extracts are continuously being tested against foodborne pathogens and spoilage agents, both in vitro and i*n situ*, to identify potent alternatives for extending the shelf life of various food products, particularly those highly susceptible to microbial contamination, such as meat, dairy, grains, and cereals. Advances in plant antimicrobial research have the potential to enhance food safety by reducing foodborne illnesses and minimize financial losses in the food industry by preventing spoilage and extending product shelf life. Additionally, these advancements could contribute to environmental sustainability by reducing food waste and promoting more efficient food production practices.

## In silico screening for plant antimicrobials

Traditionally, in vitro and in situ techniques have been used to identify antimicrobials in plant-derived extracts. However, more recently, in silico screening of databases such as DrugBank, ChEMBL, PubChem, PDB (Protein Data Bank), TTD (Therapeutic Target Database), DGIdb (Drug-Gene Interaction Database), STITCH, and STRING has been utilized to discover potential antimicrobial compounds, proving to be a fast and reliable high-throughput method (Rognan 2017). Several computational approaches, such as molecular docking and molecular dynamics simulations, have been developed to rapidly identify bioactive molecules by predicting their interactions with microbial targets.

Molecular docking is based on the interaction between a receptor (typically an enzyme) and a ligand (such as a substrate, product, inhibitor, activator, or prosthetic group), and is used to analyze the strength and specificity of their interaction, assess binding affinity, and predict the optimal structural conformation of the ligand–receptor complex (Zhang et al. 2020). Molecular docking was used to verify the antimicrobial activity of the phytochemicals from *Citrus limon* (Family: Rutaceae) leaf essential oil against *P. aeruginosa* proteins. The higher the binding affinity between the pathogen enzyme (receptor) and the plant extract compound (ligand), the more successful is the inhibition (Raji et al. 2017). Molecular dynamics simulation is a computer simulation that analyzes the atomic movements of an enzyme over a defined period, providing information about its structural function such as activity, stability, catalytic processes, and flexibility. Molecular docking combined with molecular dynamics is commonly used to discover new food enzymes based on microbial or plant genomes, to catalyze new reactions, optimize or inactivate other enzymes, or to create new enzymes for specific purposes (Rognan 2017).

Quorum sensing (QS) is a bacterial intercellular communication mechanism that regulates gene expression within a population to induce group behaviors, such as virulence and biofilm formation. By contrast, anti-quorum sensing (anti-QS) strategies aim to disrupt these bacterial actions and, consequently, control pathogenicity. As a result, there is a growing demand for anti-QS compounds to combat bacterial infections in food (Asfour 2018). An in silico experiment was conducted to screen the QS inhibitory activities of selected phytoconstituents from *Ficus* spp. extracts (Family: Moraceae). Following in vitro analysis, the most promising extracts were evaluated in anti-QS studies using molecular docking to characterize their interaction patterns. Based on the observed interactions between phytochemicals and key amino acids, it was concluded that *Ficus*-derived compounds are potential QS inhibitors and may offer effective antibacterial activity against foodborne pathogens, thereby, enhancing food preservation and extending shelf life (Elhawary et al. 2018).

High-throughput screening (HTS) is an automated process that enables biochemical events to be rapidly tested thousands of times at a large scale, to identify bioactive compounds, understand interaction patterns, and detect specific properties such as enzyme activity, antimicrobial effects, toxicity levels, or binding affinities (Tripathi and Bandyopadhyay 2022). Used extensively in the pharmaceutical industry, HTS has been gaining importance in plant antimicrobial studies. Thin layer chromatography-direct Bioautography (TLC-DB) is an HTS method that was used to detect and characterize antimicrobial and antifungal compounds in several plant samples (Choma and Jesionek 2015). Another assay used the HTS platform for the identification of a novel compound from natural sources that can either inhibit the formation or eliminate and disperse preformed biofilm by *Salmonella* (Paytubi et al. 2017). HTS is still an emerging technique for the food industry, however, it already shows great potential to investigate plant antimicrobials and has the potential to study natural preservatives in the future.

**Fig. 3:**
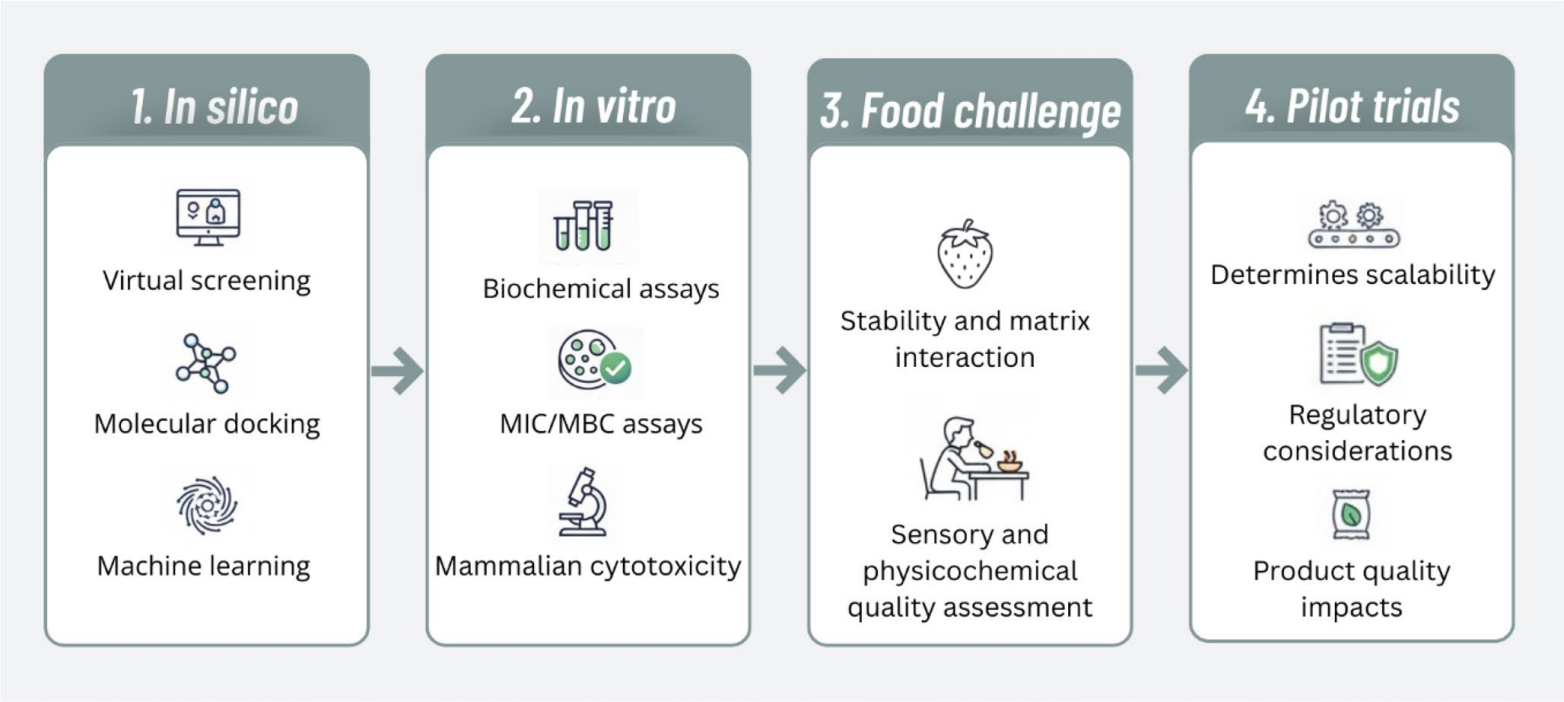
Workflow illustrating the sequential stages involved in the identification and validation of plant-derived antimicrobials, from in silico screening to in vitro assays, food challenge tests, and pilot-scale trials

The integration of computational tools into the antimicrobial development pipeline is illustrated in a schematic workflow (Fig. 3), depicting the progression from in silico screening to in vitro assays, food challenge tests, and ultimately pilot-scale studies. This workflow underscores the translational nature of the process and highlights the role of computational predictions as an initial prioritization step, supporting the selection of promising candidates and guiding subsequent experimental validation.

Although highly valuable for screening and prioritization, in silico approaches exhibit intrinsic limitations. Computational methods such as molecular docking and molecular dynamics rely on simplified representations of biological systems and therefore cannot fully capture the physicochemical, structural, and compositional complexity of real food matrices. As the influence of the matrix has been extensively demonstrated, through factors such as pH, water activity, ionic strength, protein–lipid interactions, microstructural heterogeneity, and processing-induced modifications, antimicrobial performance may differ substantially from computational expectations. Consequently, in silico outputs should be interpreted as preliminary hypotheses that require rigorous validation through in vitro assays and food-system challenge studies.

## Safety, Toxicology, and regulatory considerations

International regulatory agencies require structured safety assessment before plant-derived antimicrobials can be used in foods. In the United States, the Food and Drug Administration (FDA) evaluates food additives under the GRAS framework, supported by toxicological studies that typically include in vitro genotoxicity assays, acute toxicity tests, dose-range finding studies, and 90-day repeated-dose studies in rodents, depending on expected dietary exposure (FDA 2006). In the European Union (EU), the safety evaluation of plant-derived compounds intended for use in foods is carried out by the European Food and Safety Authority (EFSA) through a tiered risk-assessment paradigm. This process includes chemical characterization (identity, specifications, and stability), toxicokinetic assessment (absorption, distribution, metabolism, and excretion), and toxicological testing such as genotoxicity, repeated-dose (sub-chronic) toxicity, and, where relevant, reproduction and development toxicity (EFSA 2012). Expected dietary exposure is also considered and integrated with all available evidence to support a comprehensive safety conclusion (Medeleanu et al. 2024).

Several plant-derived compounds already have regulatory acceptance. In the USA, essential oil constituents such as thymol, carvacrol, eugenol, cinnamaldehyde, citral, and linalool are classified as GRAS for specific food categories, with maximum permitted levels defined in the Title 21 of the Code of Federal Regulations (CFR) based on exposure and toxicological thresholds (United States 1977). In the EU, botanical additives and flavouring substances are regulated under Regulation (EC) N^o^ 1333/2008 and evaluated by EFSA’s Food Additives and Flavourings Panel (FAF), which may assign acceptable daily intakes (ADI) or conclude that exposure falls below Threshold of Toxicological Concern (TTC) limits (Younes et al. 2021). Allergenicity is an additional regulatory concern, particularly for plant-derived proteins, such as lectins, storage proteins, and antimicrobial peptides, and is evaluated through sequence-homology screening, in vitro digestibility assays, and documented sensitization reports (Naegeli et al. 2017).

In parallel with regulatory assessments, experimental data from cellular and animal models contribute to defining the safety profile of plant-derived antimicrobials. Compounds such as thymol and carvacrol have demonstrated anti-inflammatory and antioxidant effects in vivo, reducing cytokine production and modulating NF-κB, ERK1/2, and Nrf2/HO-1 pathways in murine models of endotoxemia or inflammation (Yao et al. 2018; Yan et al. 2023). Other molecules, including plant-derived antimicrobial peptides, have also shown promising safety profiles. For example, Nicotianin I, isolated from *Nicotiana* floral nectar, exhibited fungicidal activity against *Candida* spp. without inducing cytotoxicity in human or murine cells and showed limited toxicity in zebrafish embryos (Neto et al. 2025). These findings illustrate the importance of integrating efficacy results with toxicological endpoints across multiple test systems.

A growing area of interest concerns the potential contribution of plant antimicrobials to antimicrobial resistance (AMR). Although most phytochemicals act through multiple, non-specific mechanisms, such as membrane disruption, protein denaturation, or oxidative damage, sublethal or improper exposure may potentially promote bacterial resistance via modulation of efflux pumps or stress-response pathways, similar to processes observed with antibiotics (Vranakis et al. 2014; Yang et al. 2018). To date, no food-related cases of AMR associated with plant-derived compounds have been reported, and the overall risk is considered substantially lower than that of conventional antibiotics. Nonetheless, appropriate dosing and avoidance of prolonged sub-inhibitory exposure remain essential.

## Practical applications

Recent technological advances have significantly expanded the practical applicability of plant-derived antimicrobials in foods. Encapsulation and nanoencapsulation strategies, including polymeric nanoparticles, nanoemulsions, solid lipid nanoparticles, and liposomal systems, have been widely employed to improve the chemical stability, dispersion, and controlled release of EO constituents and other phytochemicals, thereby enhancing their antimicrobial performance in complex food matrices (Albuquerque et al. 2022; Bolgen et al. 2025). The incorporation of plant-derived antimicrobials into edible coatings and films has also gained attention, particularly in polysaccharide- and protein-based systems that enable gradual release at the food surface, resulting in improved microbial control, extended shelf-life, and better quality attributes (Moradinezhad et al. 2025). Parallel developments in active packaging materials, especially bio-based polymers functionalized with natural antimicrobials or their nanoformulations, have likewise demonstrated promising outcomes in reducing spoilage and inhibiting pathogen growth (Andrade et al. 2025).

Moreover, notable synergies with non-thermal processing technologies have been widely reported. High-pressure processing enhances membrane permeability and can potentiate the antimicrobial activity of essential oils and spice extracts, thereby reducing the concentrations required for microbial inactivation (Espina et al. 2013; Xiao et al. 2021). Pulsed electric fields similarly induce electroporation and facilitate the uptake of phytochemicals by microbial cells, contributing to additive or synergistic inactivation effects (Ghoshal 2023). Cold plasma technologies further complement natural antimicrobials by generating reactive species that increase microbial susceptibility (Mahmoud et al. 2025). Additionally, the combined application of grapefruit essential oil and UV-C irradiation has been shown to enhance microbial inactivation in fresh produce, achieving 1.92–3.96 log reductions of *E. coli* O157:H7 and human norovirus surrogates, shortening the time required for complete inactivation, and maintaining antimicrobial efficacy during refrigerated storage (Liao et al. 2024).

Collectively, these applications highlight the translational potential of plant-derived antimicrobials and support their integration into industrial food preservation strategies.

## Conclusion

Plant-derived antimicrobials represent a promising and sustainable alternative to synthetic preservatives for controlling foodborne pathogens and spoilage microorganisms. The wide array of bioactive compounds found in medicinal and aromatic plants, such as phenolics, alkaloids, essential oils, lectins, and antimicrobial peptides, has shown significant efficacy across various food matrices. Advances in extraction, purification, and characterization techniques, including innovative methods like ultrasound-assisted extraction and pressurized liquid extraction, along with high-resolution analytical tools (e.g., HPLC, GC-MS, NMR), have enhanced the efficiency and precision in isolating these compounds. Additionally, in silico screening approaches, such as molecular docking and molecular dynamics simulations, have facilitated the discovery and mechanistic understanding of plant antimicrobials, accelerating the identification of candidates for food preservation. Despite their promising potential, further research is required to assess the safety, stability, and effectiveness of these compounds under industrial processing conditions, as well as their interactions within complex food systems. The incorporation of plant-derived antimicrobials into food preservation strategies not only meets consumer demand for natural ingredients but also contributes to improved food safety, extended shelf-life, and reduced food waste.
